# Portable Raman Spectrometer as a Screening Tool for Characterization of Iberian Dry-Cured Ham

**DOI:** 10.3390/foods10061177

**Published:** 2021-05-24

**Authors:** Andrés Martín-Gómez, Natalia Arroyo-Manzanares, María García-Nicolás, Ángela I. López-Lorente, Soledad Cárdenas, Ignacio López-García, Pilar Viñas, Manuel Hernández-Córdoba, Lourdes Arce

**Affiliations:** 1 Department of Analytical Chemistry, Institute of Fine Chemistry and Nanochemistry, Marie Curie Annex Building, Campus de Rabanales, 14071 Córdoba, Spain; q02magoa@uco.es (A.M.-G.); q32loloa@uco.es (Á.I.L.-L.); qa1caarm@uco.es (S.C.); qa1arjil@uco.es (L.A.); 2 Department of Analytical Chemistry, Faculty of Chemistry, Regional Campus of International Excellence “Campus Mare Nostrum”, University of Murcia, E-30100 Murcia, Spain; maria.garcia66@um.es (M.G.-N.); ilgarcia@um.es (I.L.-G.); pilarvi@um.es (P.V.); hcordoba@um.es (M.H.-C.)

**Keywords:** authentication, screening, Iberian ham, Raman

## Abstract

Dry-cured Iberian ham is officially classified into different commercial categories according to the pig’s breed and feeding regime. These reach very different prices, thus promoting labelling fraud and causing great damage to the food sector. In this work, a method based on Raman spectroscopy was explored as a rapid in situ screening tool for Iberian ham samples. A total of 110 samples were analyzed to assess the potential of this technique to differentiate purebred, crossbred, acorn-fed and feed-fed dry-cured Iberian ham. A continuous signal probably due to sample fluorescence was obtained, which hid the Raman scattering signal. Therefore, chemometric treatment was applied in order to extract non-apparent information. High validated classification rates were obtained for feeding regime (83.3%) and breed (86.7%). In addition, an interlaboratory study was carried out to confirm the applicability of the method with 52 samples, obtaining a validated rate above 80%.

## 1. Introduction

Acorn-fed or bellota dry-cured Iberian ham is a product derived from Iberian pigs fattened up by grazing acorns and pasture in a free-range or montanera regime. It is the most valued animal product from the dehesa agri-ecosystem. Its high commercial value has led to an increase in labelling fraud, both in terms of the pig feeding regime and racial purity. To overcome this problem, recently, the Spanish “Iberian Pork Quality Regulation” [1] was published. While this regulation set the criteria to classify Iberian ham in different commercial categories, it did not include any analytical method for its authentication. Since its implementation, the annual production of “acorn-fed” Iberian hams has increased drastically. However, neither the extensive livestock nor the acorn-producing dehesa area were multiplied, which could be a sign of possible fraud in the Iberian ham sector. For this reason, a standardized analytical method could help the authorities to fight fraud and provide guarantees to the final consumer. Several years ago, the sector accepted that the Iberian pig feeding regime could be verified by analyzing the subcutaneous fat fatty acids using gas chromatography (GC) coupled with a flame ionization detector (FID) [2]. This method condensed the value of the montanera regime to a mere fatty acid profile. At present, this profile can be easily achieved with feeds formulated for that purpose. Consequently, the current official method does not allow us to discern between a true montanera finishing or feed-feeding. This background has led to the search for new analytical methods to discriminate between Iberian hams with differing qualities.

In recent years, dry-cured Iberian ham authentication has been attempted with well-studied techniques, such as conventional gas chromatography–mass spectrometry (GC-MS) [3,4] or the more advanced ultra-high-resolution mass spectrometry (UHRMS) [5]. In recent times, gas chromatography–ion mobility spectrometry (GC-IMS) has been explored for the analysis of ham slices [6] and cured fat [7], focusing on classification. On the other hand, faster analytical techniques have been successfully employed, which include IMS without a previous separation [8]. In this case, raw pig fat was analyzed in order to differentiate ham samples of pigs fed with fodder that emulate a free-range regime from samples of pigs fed within a genuine extensive system. Furthermore, a fast and non-destructive technique, near-infrared spectroscopy (NIRS), has been studied as a tool for Iberian pig carcass classification according to feeding regime [9,10,11,12].

Among the techniques already used for the authentication of dry-cured meat, the faster ones, such as NIRS, are preferred by the stakeholder, so a similar technique in terms of fast response was studied in this project. Raman spectroscopy has been broadly used in studies of porcine meat quality control. Its applications comprise the characterization of raw fat [13,14], boar taint detection [15], determination of sensory properties [16], pH [17], ageing [18,19], spoilage [20] and more relevantly, the detection of undeclared mixes with meat from different species [21,22,23]. As depicted, while Raman spectroscopy has been employed as a tool for the quality control of raw meat and fat [24], it has no current application for the authentication of dry-cured meat. On this basis, in the present paper, one of the most representative meat products from the Iberian pig was analyzed with a Raman spectrometer: its dry-cured ham. Its four commercial categories (acorn-fed purebred Iberian, acorn-fed crossbred Iberian, free-range feed-fed crossbred Iberian and feed-fed crossbred Iberian) share similar characteristics and composition. Hence, its classification is complex, requiring data pre-treatment and chemometric approaches. Therefore, the first objective of the present paper was to demonstrate the potential of a conventional Raman and portable device as a screening technique for dry-cured Iberian ham classification. The second objective was to ensure the robustness of the method with an interlaboratory study, comparing the classification results of two similar Raman devices.

## 2. Materials and Methods

### 2.1. Samples

A total of 162 samples were analyzed. The first set consisted of 110 samples (48 samples of 100% Iberian acorn-fed, 32 samples of 50–75% Iberian acorn-fed and 30 samples of 50–75% Iberian feed-fed dry-cured hams). The second set consisted of 52 samples (22 samples of 100% Iberian acorn-fed, 8 samples of 75% Iberian feed-fed and 22 samples of 50% Iberian feed-fed dry-cured hams). The first set of samples was analyzed using device A and the second set using device B.

Samples of feed-fed ham were produced in different parts of Spain and purchased in local markets or supplied by the protected designation of origin (PDO) company Jamones Ibéricos Dehesa de Campo Alto S.L. (Espiel, Córdoba, Spain). Acorn-fed samples were supplied by four companies from Los Pedroches (Córdoba) PDO and others from Salamanca, Zaragoza, Huelva and Badajoz.

All samples were extracted from the ham cushion (“maza”) and stored at −4 °C until their analysis (2–3 days after collection) to prevent lipid oxidation. The procedure of sample preparation was standardized: samples were sliced, cut into a single portion of approximately 1 mm in thickness and placed over aluminum foil to be analyzed by Raman spectroscopy. Among the advantages of this sampling method is that weighing the sample is not necessary.

### 2.2. Instruments and Software

Raman spectra measurements of the samples were performed with two similar i-Raman BWS415 portable spectrometer systems acquired from B&W Tek Europe GmbH (Lübeck, Germany) using a laser with a wavelength of 785 nm and a maximum laser output power at the system’s excitation port of 354 mW ± 15% and 285 mW in the probe. The devices were located in two different laboratories in Spain (device A was located at University of Córdoba and device B at University of Murcia). Spectra were obtained in the range of 1–2035 cm^−1^ with a resolution of 4.5 cm^−1^. For the measurements, the laser power at the probe was set to 10% of the maximum, which was 28.5 mW. The laser beam was focused on the sample through a 20×/0.40 NA objective. Spectra were recorded using a 500 ms acquisition time, a time average of 3 and a multiplier of 10. Data were collected by using BWSpec 3.27 software.

MATLAB software (The Mathworks Inc., Natick, MA, USA, 2007) and PLS Toolbox 5.5 (Eigenvector Research, Inc., Manson, WA, USA) were used for data processing.

### 2.3. Data Processing

**Figure 1:**
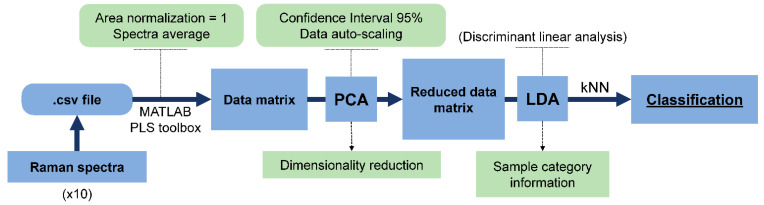
Data processing scheme of Raman spectra.

The obtained spectral data were exported to a csv file after the substraction of the dark spectra, taken with no light reaching the detector. A total of 10 different superficial points of the samples were analyzed, so 10 spectra were obtained from each sample. Initially, a matrix was built for each sample (dimension 10 × 2048). Afterwards, each matrix was normalized (1-Norm = Area 1) and mean-centered using PLS Toolbox 5.5. Subsequently, the averaging of the 10 spectra measured for each sample was performed using MATLAB. Then, a new matrix composed of the average spectra of all the ham samples, each one placed in a row, was used for chemometric analysis. This matrix was randomly split into two datasets: 80% of samples for construction of the chemometric models (training set) and 20% of samples for model validation (validation set). Then, a non-supervised PCA analysis using auto-scaled data followed by linear discriminant analysis (LDA) was carried out. LDA was used as a supervised linear projection technique to incorporate class information into the model and ascertain if the samples were grouped in separated clusters. Finally, the k-nearest neighbor method (kNN) using k = 3 was applied to the validation set in order to obtain the percentage of correctly classified samples. Data processing steps are summarized in Figure 1. This process was repeated 5 times, selecting new validation and training sets at random, in order to check the robustness of the results.

## 3. Results and Discussion

### 3.1. Method Optimization

**Figure 2:**
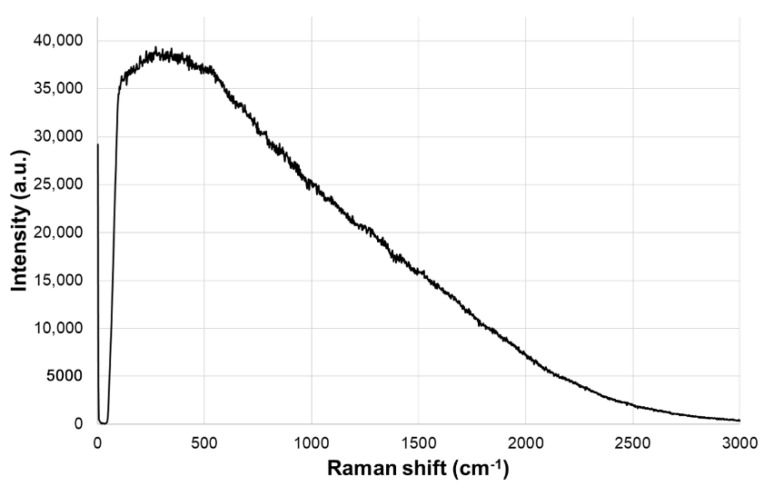
Spectra obtained for an Iberian ham sample, dominated by the fluorescence profile.

When using Raman spectroscopy to analyze dry-cured Iberian ham, a continuous signal probably due to sample fluorescence was obtained, which hid the Raman scattering signal (Figure 2). Consequently, a conventional approach through the identification of Raman bands could not be followed. Several papers have been devoted to finding a method to suppress the fluorescence background in Raman measures [25]. Some of them were based on data treatment, such as polynomial curve fitting [26] or algorithms based on Savitzky–Golay [27]. Furthermore, standard normal variate (SNV) and multiplicative scatter correction (MSC) have been applied for biological samples [28,29]. On the other hand, unconventional Raman techniques have also been employed, such as shifted-excitation Raman difference spectroscopy (SERDS) [30]. This technique allows the location of Raman bands among extreme fluorescence. However, in this work, instead of moving efforts to find the best method to eliminate the Raman background, a direct sample classification of the spectral data was attempted.

The instrumental parameters were optimized to improve the results according to the objective of this work. The laser power was optimized from 285 mW to the minimum reachable (28.5 mW), as an excessive power could burn the ham surface. In order to obtain a representative average spectrum, the number of measurements of each sample was also studied. Subsequently, it was demonstrated that it was necessary to record 10 Raman spectra at different points of the sample to collect all its variability.

### 3.2. Classification of Dry-Cured Hams

The application of chemometrics for Raman spectra in this study was newly developed due to difficulties interpreting individual band signals. As previously mentioned, a fluorescence background obscures the Raman scattering signal in dry-cured Iberian ham samples. Therefore, a non-target classification without the identification of Raman bands was attempted. Supplemental Figure S1 shows spectra from each ham category, and as can be seen, visual differentiation was not possible.

Initially, data pre-processing was necessary before the construction of the chemometric models. This pre-processing included a normalization step for each spectrum. Different normalization approaches were investigated (respect to height or area) obtaining the best result using the area. Once the data were normalized, the averaging of the 10 spectra taken from each sample was performed. Consequently, each sample would be represented by its average spectrum.

The first set of samples (48 samples of 100% Iberian acorn-fed, 32 samples of 50–75% Iberian acorn-fed and 30 samples of 50–75% Iberian feed-fed dry-cured ham) was analyzed using device A and the proposed methodology. After data pre-processing, a matrix with dimensions of 110 × 2048 was obtained. 

Initially, a ternary model to classify the three types of ham samples was investigated. However, validation rates below 50% were obtained, and the use of two sequential classification models was proposed.

**Figure 3:**
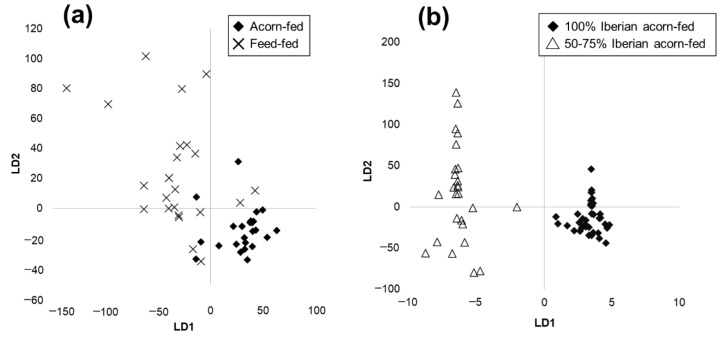
Score plots of LDA calibration models for (**a**) feeding regime and (**b**) breed discrimination using device A.

The first chemometric binary model was aimed at classification by feeding regime. In order to compensate the groups, 16 samples of 100% Iberian acorn-fed and 16 samples of 50–75% Iberian acorn-fed formed the acorn-fed group, and 30 samples of 50–75% Iberian feed-fed formed the feed-fed group. The model was built using 80% of the ham samples and validated with the remaining 20%. A PCA allowed us to reduce the dimensionality to 20 principal components (99% cumulative variance). Subsequently, an LDA calibration model was obtained, whose score plot is shown in Figure 3a. Afterwards, a kNN algorithm with k = 3 was applied to the scores of the validation set in order to obtain the percentage of correctly classified samples. These models were repeated using an average of five spectra for each sample and using all the spectra acquired from each sample contained in a single file. However, the best validation ratio was obtained when a sample was represented by the average of its 10 spectra. Table 1 shows the results obtained from this binary model, and as can be seen, high validated classification rates were obtained for feeding regime, with a success in the classification of 83.3% for both classes.

The second model was aimed at the classification by breed of acorn-fed dry-cured ham. In this case, a total of 48 of 100% Iberian acorn-fed and 32 of 50–75% Iberian acorn-fed samples were employed. The chemometric model was also built using 80% of the ham samples and validated with the remaining 20%. As in the first model, the PCA reduced the dimensionality to 20 principal components (99% cumulative variance). Afterwards, an LDA calibration model was obtained, whose score plot is shown in Figure 3b. Subsequently, a kNN algorithm with k = 3 was applied to the scores of the validation set to obtain the percentage of correctly classified samples. As may be noted in Table 2, high validated classification rates were also achieved for breed, with a success in the classification of 77.8 and 100% for purebred and crossbred samples, respectively. This involves a total success rate of 86.7%.

**Figure 4:**
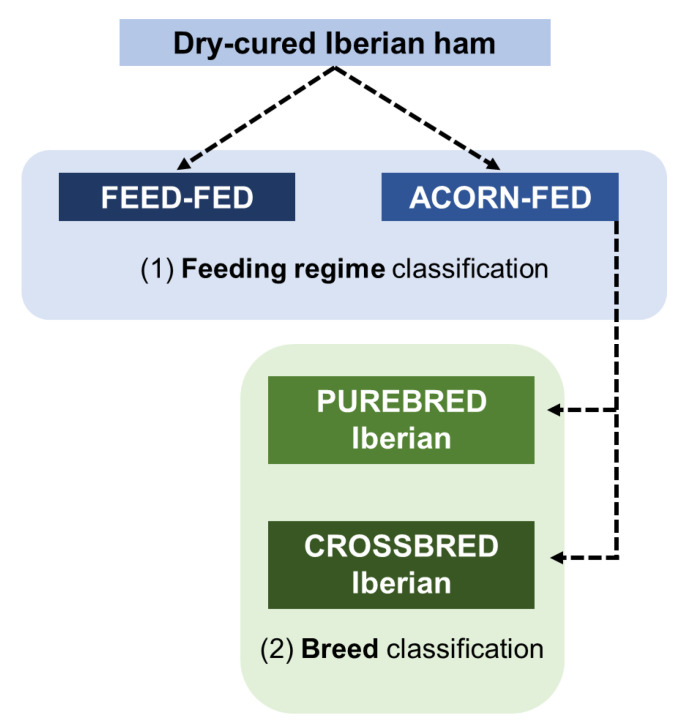
Procedure for Iberian ham classification in the industrial setting using the developed Raman method.

These results demonstrate that the developed Raman method could be applied in the industrial setting to carry out a screening analysis. The procedure would consist of applying a first chemometric model for feeding regime classification. Afterwards, a breed chemometric model would isolate the purebred Iberian pieces that have the highest quality and commercial value. The procedure is summarized in Figure 4.

### 3.3. Interlaboratory Study

**Figure 5:**
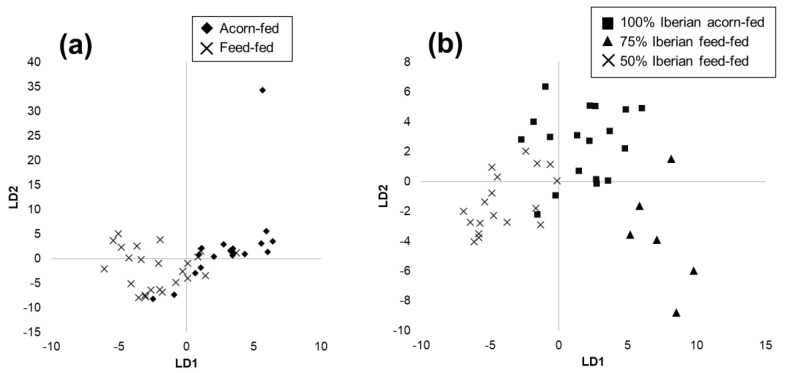
Score plots of LDA calibration models for (**a**) feeding regime discrimination and (**b**) ternary differentiation using device B.

In order to assess the robustness of the proposed analytical method, a second set of samples (22 samples of 100% Iberian acorn-fed, 8 samples of 75% Iberian feed-fed and 22 samples of 50% Iberian feed-fed dry-cured ham) was analyzed using Raman device B following the previously described methodology. After data pre-processing, a matrix with dimensions of 52 × 2048 was obtained. Subsequently, two PCA-LDA chemometric models were developed. A binary chemometric model was also built for feeding regime discrimination (acorn-fed or feed-fed) using 80% of the ham samples (18 samples of 100% Iberian acorn-fed and 24 samples of 50–75% Iberian feed-fed) and validated with the remaining 20% (4 of 100% Iberian acorn-fed and 6 of 50–75% Iberian feed-fed). The PCA allowed us to reduce the dimensionality to 10 principal components (99% cumulative variance). As in the procedure of Section 3.2, an LDA was then carried out, and a kNN algorithm with k = 3 was applied to the validation set in order to obtain the corresponding classification rates. The developed model provided acceptable results as can be inferred from the score plot in Figure 5a. Accordingly, it was successfully employed to classify the validation set. An excellent classification rate was obtained for acorn-fed samples (100%), as observable in Table 3, which includes the validation matrix. On the other hand, the classification rate for feed-fed samples was not as high (50.0%), which reduced the total success in the classification of the feeding regime model to 70.0%. Interestingly, while the feed-fed samples analyzed with device A came from Iberian pigs strictly fed with fodder in an intensive regime, the feed-fed samples analyzed with device B came from pigs fed in an extensive regime with a broader mix of fodder and pasture (which are officially designated as “Cebo de campo”). In this case, the diet may even occasionally include acorns. Hence, this fact could explain the small variation in the validation rate between both devices.

Unfortunately, 50–75% Iberian acorn-fed samples with good traceability were not available to be analyzed with device B; thus, the binary model to classify acorn-fed samples by breed could not be carried out. On the contrary, in this set of samples, there was information available to differentiate between 50 and 75% Iberian feed-fed samples. Therefore, we decided to build an additional ternary model for a simultaneous classification according to feeding regime and breed (100% Iberian acorn-fed, 75% Iberian feed-fed or 50% Iberian feed-fed). This chemometric model was built using 80% of the ham samples (18 of 100% Iberian acorn-fed, 6 of 75% Iberian free-range feed-fed and 18 of 50% Iberian free-range feed-fed) and validated with the remaining 20% (4 of 100% Iberian acorn-fed, 2 of 75% Iberian free-range feed-fed and 4 of 50% Iberian free-range feed-fed). Subsequently, a PCA was carried out, and the dimensionality was reduced to 10 principal components (99% cumulative variance). Afterwards, an LDA was carried out, and a kNN algorithm with k = 3 was applied to the validation scores to obtain the corresponding classification rates. The ternary model provided great results, as shown in the score plot (Figure 5b), and it was successfully employed to classify the validation set. High classification rates were obtained for the three sample classes, ranging from 75 to 100%, as can be seen in Table 4, with a total success of 90.0%. However, due to the differences in the information available from both sets of samples, this ternary model could not be tested with device A.

In general terms, the validated classification rates provided by devices A and B were similar. The obtained results demonstrate the suitability and robustness of Raman analysis for the authentication of Iberian dry-cured ham, despite the absence of conventional bands on the spectra.

## 4. Conclusions

The displayed results demonstrated that Raman spectroscopy could be used as a fast in situ screening tool to authenticate the quality of commercial dry-cured Iberian ham. A first Raman instrument was employed to classify 110 samples of dry-cured Iberian ham obtained from pigs with different breed purities and feeding regimes. The LDA chemometric models obtained with the Raman signal allowed a categorization according to the pig’s breed and feeding regime with no need for the isolation of additional bands. This approach was employed for the first time in dry-cured meat. Moreover, an interlaboratory study carried out with an additional Raman device and the analysis of 52 additional samples ensured the robustness of the validated percentages of classification, which on average ranged between a promising 80.0–90.0% of success. In the near future, Raman spectroscopy could serve as a useful screening technique for fast in situ analyses (5 min approximately) for the detection of labelling fraud within this product with high added value. Despite the usefulness demonstrated, to ensure the applicability of the methods in routine analysis, further studies could be necessary.

## Figures and Tables

**Table 1:** Results of feeding regime Raman chemometric classification model with device A.

Feeding Regime		Total	% Correctly Predicted	Predicted Classes		
				Acorn-Fed	Feed-Fed	
Actual classes	Acorn-fed	6	83.3	5	1	Success 83.3%
	Feed-fed	6	83.3	1	5	

**Table 2:** Results of breed Raman chemometric classification model with device A.

Breed		Total	% Correctly Predicted	Predicted Classes		
				100% Iberian	50–75% Iberian	
Actual classes	100% Iberian	9	77.8	7	2	Success 86.7%
	50–75% Iberian	6	100	0	6	

**Table 3:** Results of feeding regime Raman chemometric classification model with device B.

Feeding Regime		Total	% Correctly Predicted	Predicted Classes		
				Acorn-Fed	Feed-Fed	
Actual classes	Acorn-fed	4	100	4	0	Success 70.0%
	Feed-fed	6	50.0	3	3	

**Table 4:** Results of ternary Raman chemometric classification model with device B.

Ternary		Total	% Correctly Predicted	Predicted Classes			
				100% Iberian Acorn-Fed	75% Iberian Feed-Fed	50% Iberian Feed-Fed	
Actual classes	100% Iberian acorn-fed	4	100	4	0	0	Success 90.0%
	75% Iberian feed-fed	2	100	0	2	0	
	50% Iberian feed-fed	4	75	1	0	3	

## Supplementary Materials

The following are available online at https://www.mdpi.com/article/10.3390/foods10061177/s1, Figure S1: Spectra from the different samples categories: (A) 50% Iberian acorn-fed, (B) 100% Iberian acorn-fed, (C) 75% Iberian feed-fed. Figure S2: Laser power optimization.
