# Supplementary material for: Portable Raman Spectrometer as a Screening Tool for Characterization of Iberian Dry-Cured Ham
Source: Foods. 2021 May 24;10(6):1177. doi: 10.3390/foods10061177 (PMC8225093; doi:10.3390/foods10061177)
Supplement: Supplementary file 1 [file foods-10-01177-s001.zip › foods-1219513-supplementary.pdf]

foods

Supplementary Material

Portable Raman spectrometer as a screening tool for characterization of Iberian dry-cured ham

A. Martín-Gómez[1], N. Arroyo-Manzanares[2*], M. García-Nicolás[2], A.I. López-Lorente[1], S. Cárdenas[1], I. López-García[2], P. Viñas[2], M. Hernández-Córdoba[2] and L. Arce[1]

[1] Department of Analytical Chemistry, Institute of Fine Chemistry and Nanochemistry, Marie Curie Annex Building, Campus de Rabanales, 14071 Córdoba, Spain; q02magoa@uco.es (A.M.-G); q32loloa@uco.es (A.I.L.-L); qa1caarm@um.es (S.C.); qa1arjil@um.es (L.A.)

[2] Department of Analytical Chemistry, Faculty of Chemistry, Regional Campus of International Excellence "Campus Mare Nostrum", University of Murcia, E-30100 Murcia, Spain; maria.garcia66@um.es (M.G.-N.); ilgarcia@um.es (I.L-G.); pilarvi@um.es (P.V.); hcordoba@um.es (M.H.C.)

* Correspondence: natalia.arroyo@um.es (N.A-M.)

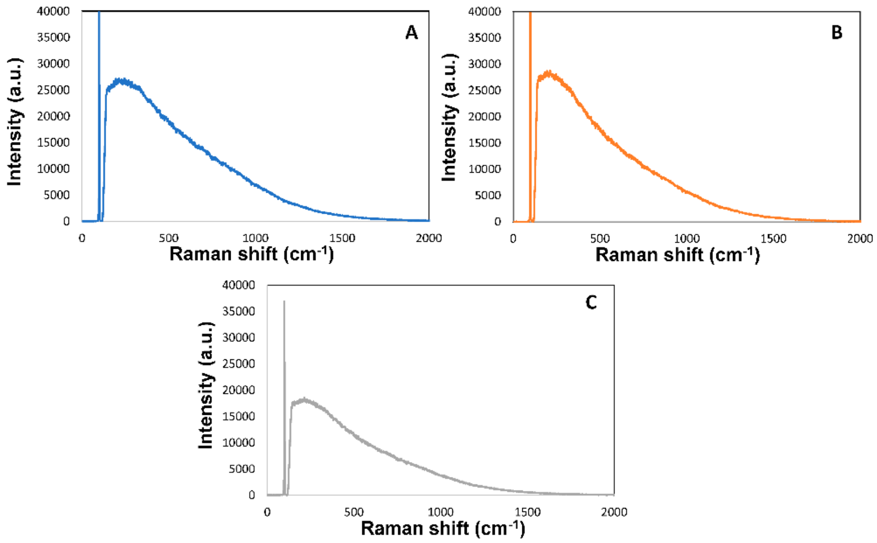

Supplemental Figure S1. Spectra from the different samples categories: A) 50% Iberian acorn-fed, B) 100% Iberian acorn-fed, C) 75% Iberian feed-fed.

foods MDPI

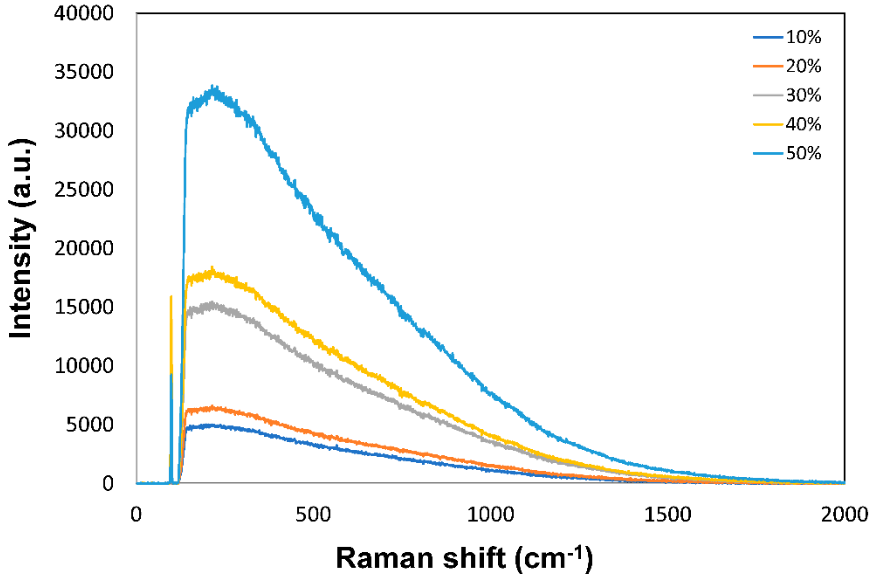

Supplemental Figure S2. Laser power optimization.
